# Nanosorbents as Materials for Extraction Processes of Environmental Contaminants and Others

**DOI:** 10.3390/molecules27031067

**Published:** 2022-02-05

**Authors:** María José Santoyo Treviño, Sergio Zarazúa, Justyna Płotka-Wasylka

**Affiliations:** 1 Laboratory of Neurotoxicology, Faculty of Chemistry, Autonomous University of San Luis Potosí, Av. Manuel Nava 6, Zona Universitaria, San Luis Potosí 78210, Mexico; mjst1191@gmail.com (M.J.S.T.); sergio.zarazua@uaslp.mx (S.Z.); 2 Department of Analytical Chemistry, Faculty of Chemistry and BioTechMed Center, Gdańsk University of Technology, 11/12 G. Narutowicza St., 80-233 Gdańsk, Poland

**Keywords:** nanomaterials, nanoparticles, extraction techniques, sorbent, sample preparation

## Abstract

The aim of this work focuses on the application of nanomaterials (NMs) in different sorptive extraction techniques for the analysis of organic contaminants from environmental samples of distinct matrix compositions. Without any doubt, the integration of specific NMs such as carbonaceous nanomaterials, magnetic nanoparticles (MNPs), metal–organic frameworks (MOFs), silica nanoparticles, and ion-imprinted NPs with solid-phase extraction techniques counting *d*-SPE, solid-phase microextraction (SPME), and stir bar sorptive extraction (SBSE) impact on the improvements in analytical performance. The application of NMs as sorbents in the extraction of organic pollutants in environmental samples allows for providing better sensitivity, repeatability, reproducibility, and reusability.

## 1. Introduction

Much research on the application of nanomaterials in different scientific and industrial fields is being performed in areas such as oil processing, sensors, water treatment, building materials, catalysis, food, and construction [1]. NMs have also become an object of interest in the field of analytical chemistry activities [2]. Over the last two decades, NMs have been widely applied as sorbents for the extraction of environmental contaminants [2]. This is due to their unique properties such as large surface area and fast adsorption capability, and they also present high selectivity and efficiency for environmental pollutants. The most popular NMs used as sorbents for these purposes are magnetic nanoparticles (MNPs), nano-based metal–organic frameworks (N-MOFs), silica nanoparticles (SiNPs), carbon nanomaterials (CNMs), and nano-imprinted polymers (NIPs). These NMs are utilized for the isolation and pre-concentration of environmental pollutants in such techniques as solid-phase extraction (SPE), solid-phase microextraction (SPME), magnetic solid-phase extraction (MSPE), and dispersive solid-phase extraction (DSPE). It is worth mentioning that the application of these nanomaterial sorbents impacts the extraction efficiency of these techniques.

This review summarizes the basic features of analytical options that can be used for the extraction and preconcentration of pollutants in environmental samples based on the integration amongst different kinds of NMs and several types of microextraction techniques. In addition, environmental samples characterized by different matrix compositions are considered. The issue of the article focusing on the application of nanosorbents as materials for extraction processes was highly treated in the literature [3,4,5,6]. However, this review contains essential and the most significant information for researchers who deal with the complex issues connected with environmental samples and can be an easy start for future researchers in this area. In the present article, future authors will find not only information on the types of NMs applied in sample preparation processes, but also general knowledge on the specific solid-phase extraction techniques, which can be very useful for basic research. Reaxys database from Elsevier was used to find data and information necessary to perform this research, saving time and ensuring completeness of results.

## 2. Classification of Nanomaterials as Sorbents

**Figure 1:**
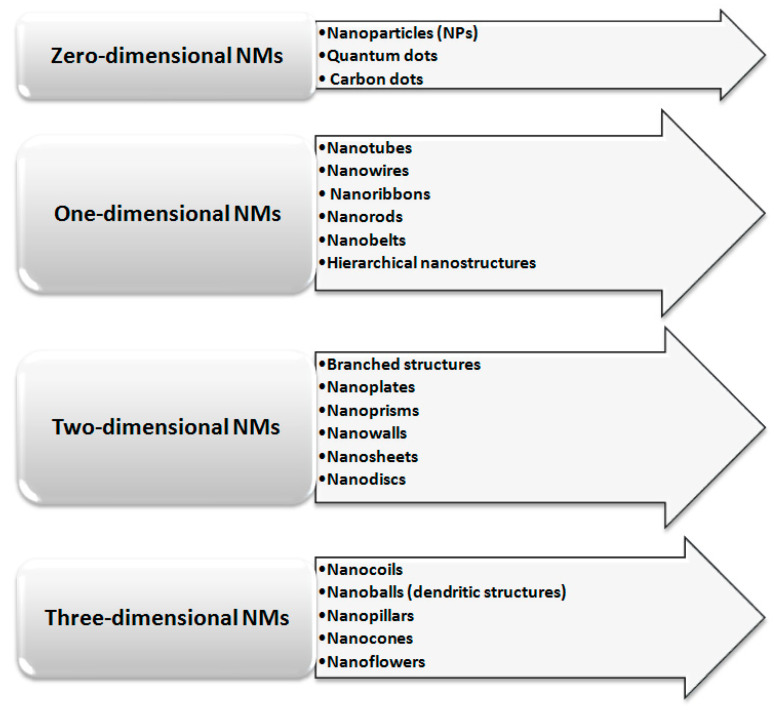
Classification of nanomaterials based on the dimensionality of materials.

The classification of NMs depends on the considered parameter, and therefore, they can be classified based on their structural geometry and chemical composition but also based on the dimensionality of materials. Considering the last parameter, NMs can be divided into (i) zero-dimensional (0–D), (ii) one-dimensional (1–D), (iii) two-dimensional (2–D), and (iv) three-dimensional (3–D). A schematic representation of this classification, together with examples, is presented in Figure 1. A given nanomaterial may belong to different groups mentioned above. Information on NMs classified in different classes is presented in Table 1.

## 3. Nanomaterials as Sorbents for Extraction of Organic Environmental Contaminants

Microextraction techniques can be a valuable tool to detect environmental contaminants in trace levels in a wide variety of matrixes such as soil, wastewater, and air. These techniques can be applied to the determination of different analytes in environmental samples such as pesticides, volatile organic compounds (VOCs), phthalates, polycyclic aromatic hydrocarbons (PAHs), and flame retardants.

Solid-phase extraction is the most frequently used technique in pre-concentration, extraction, and clean-up procedures for the identification and quantification of trace pollutants and xenobiotics [15]. This technique has a wide variety of clinical and environmental applications. However, these technologies have their limitations because they have to be used only with organic solvents due to their simplicity [16,17].

**Figure 2:**
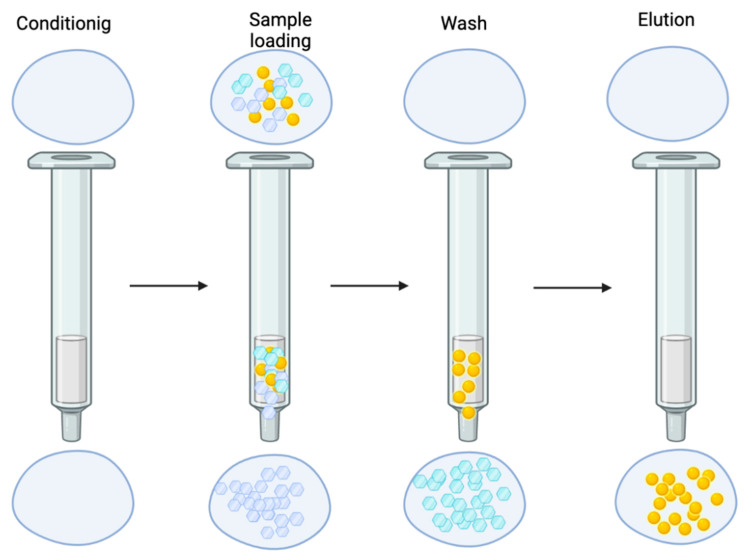
SPE consists of four critical steps, in which a solid sorbent is pre-conditioned with an appropriate solvent to remove possible impurities, wetting the packed material, and solvation of the functional groups. 1—The conditioning solvent must be selected depending on the nature of the solid sorbent; 2—The second step consists of loading the sample through the solid sorbent. 3—The third step is the optional washing of the sorbent to eliminate the residual compounds retained by the sorbent; 4—The final step involves the elution of the analytes of interest with an appropriate solvent.

The SPE procedure consists of loading a sample solution into a solid phase, which can be a cartridge with a sorbent capable of retaining target analytes and washing away undesired sample components. The complete procedure is shown in Figure 2.

According to Pole [17], SPE sorbents can be divided into 3 main groups, which are shown in Table 2.

In recent years, solid-phase microextraction (SPME) has attracted special analytical attention due to its high selectivity on trace target analytes [20]. SPME is an efficient technique integrating many operations from the extraction procedure, including the isolation of the analyte from sample matrices, sample collection, and extraction in a simple step [21]. This technique is based on the partition equilibrium principle of the target analytes among the matrix and the solid extraction phase. However, SPME has a simple and solventless sample preparation procedure [22]. The sample is exposed to a fused silica fiber coated with a layer of absorbent as the extracting phase that is directly exposed to the sample matrix for a specific amount of time until it reaches the equilibrium state [23]. SPME can be performed in three types [24], which are described in Table 3.

In comparison to SPE, SPME is characterized by simplicity, effective cleanup, less solvent consumption, higher extraction efficiency, and higher limit of detection. In addition, it is easily compatible with different instrumental techniques, especially with GC–MS. All of these advantages make SPME a perfect extraction technique used for the extraction of trace organic and inorganic analytes from different complexed environmental matrices [2]. Without any doubt, the choice of appropriate sorbents is the key role in the extraction of specific analytes. There are many commercially available sorbents; however, nanomaterials are often involved in SPME due to its special characteristics, mainly because of the large surface area and unique mechanical, thermal, magnetic, and electronic properties. It is well known that the larger surface area impacts on the increase in the interaction or adsorption capacity of the target compounds with the NPs, which results in a high capacity of extraction and efficiency for the specific analytes [25].

Depending on the analytes’ nature, a high number of nanosorbents can be selected for SPME, which are summarized and described in Table 4.

In one example, Shirani et al. developed an overhead rotating flat surface sorbent-based solid-phase microextraction for a rapid, efficient, and simultaneous separation and determination of sulfonamides in fish, cow milk, chicken meat, and egg. Three-dimensional graphene oxide/lanthanum nanoparticles @ Ni foam was introduced as a novel selective sorbent. The method is characterized by the following advantages: rapid extraction process, facile separation of the sorbent from the media, low limit of detection, and appropriate precision and accuracy [26]. The analysis of the selected food samples was performed with satisfactory relative recoveries (90.0–99.8%) and a relative standard deviation (RSD, %) less than 3.8, which is another benefit of the proposed procedure. The overall validation parameters confirm the superior potential of the methodology for application in samples characterized by complex matrices composition.

In another research study [27], SPME with polysulfone and molecularly imprinted polymers (MIPs) as a coating on Ni foam were applied to adsorb and enrich floxacin drugs from environmental water samples. The preparation procedure is simple and the coating is stable. In addition, the controlled thickness of materials is reproducible. The polysulfone did not reduce the selectivity of MIP but had the assistant function in addition to increasing the adsorption amount. Coupled with HPLC-UV-VIS analysis, the methodology is characterized by satisfied validation parameters (LOD: 3.0–6.2 μg L^−1^), as well as recoveries (90.0–104.8%), with a RSD (%) ranging from 1.0% to 9.9%.

**Table 4:** Types of nanosorbents used in SPME.

Analyte	NPs and Its Modification	Matrices	Extraction Technique	Separation Technique	LOD	Ref.
Cu, Co, Hg	Fe_3_O_4_@SiO_2_@g-MAPS	Fish, shrimp	MR/IT-SPME	HPLC-DAD	0.69–4.9 μg L^−1^	[28]
Sulfonamides	GO-La NPs @ Ni foam	chicken meat, cow meat, cow milk	RFS-SPME	HPLC-DAD	0.08-0.14 µg L^−1^	[26]
Pd, Cd	MMWCNTs-PT	Tea, milk, rice	MSPME	FAAS	0.54, 0.03 μg L^−1^	[29]
Ofloxacin drugs	OFL MIP	Water	MIP-SPME	HPLC-UV-VIS	3.0–6.2 μg L^−1^	[27]
Mo	AgPSrici	Water	DSPME	ETAAS	0.02 μg L^−1^	[30]
Cd	Co Fe_3_O_4_	Oyster	MSPME	FAAS	0.24 μg L^−1^	[31]
Cu, Pb, Cr	MDETAGOs	Juice, rice	MDSPME	DPV	0.15 ng mL^−1^	[32]

AgPSrici, polystyrene-polyricinoleic acid copolymer containing silver nanoparticles; DPV, differential pulse voltammetry; DSPME, dispersive solid-phase microextraction; ETAAS, electrothermal atomic absorption spectrometry; Fe_3_O_4_@SiO_2_@g-MAPS, Fe_3_O_4_-nanoparticles modified with tetraethylorthosilicate and 3-(trimethoxysilyl) propylmethacrylate; GO-La NPs@Ni foam, 3D graphene oxide/lanthanum nanoparticles @ Ni foam; MDETAGOs, magnetic diethylenetriamine-functionalized graphene oxide nanocomposites; MDSPME, magnetic dispersive solid-phase microextraction; MMWCNTs-PT, magnetic multi-walled carbon nanotubes modified with polythiophene; MR/IT-SPME, magnetism-reinforced in-tube solid-phase microextraction; MSPME, magnetic solid-phase microextraction; OFL MIP, ofloxacin molecularly imprinted polymers; RFS-SPME, rotating flat surface solid-phase microextraction.

**Figure 3:**
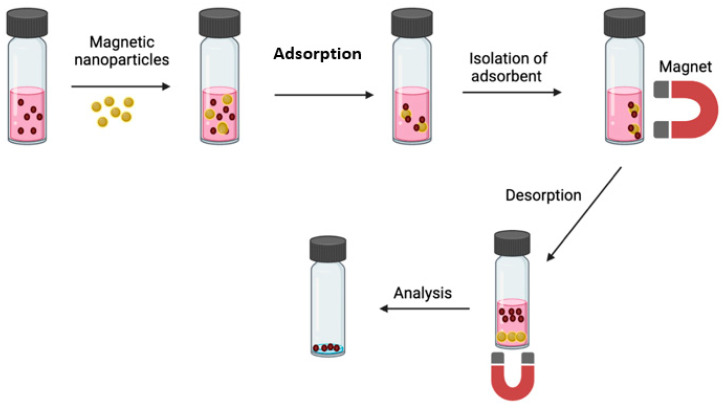
A sorbent is placed in the solution containing the target analytes. The analytes have direct contact with the sorbent NPs, which causes selective adsorption on the solid surface of the sorbent. A magnetic field in the solution is used to separate the main analyte from the solution.

In some cases, it is not possible to obtain satisfactory recovery of some trace analytes. Magnetic nanoparticles (MNPs) might be a better alternative to conventional SPE or SPME sorbents to reduce these limitations. Due to their small size, these MNPs have a good adsorption capacity and high selectivity for some analytes. The MSPE procedure is described in Figure 3.

MNPs are composed of a magnetic core made of iron, cobalt, nickel, and their oxides such as Fe_3_O_4_ [33]. The magnetic core of MNPs is not fully selective. To overcome these difficulties, the MNPs are covered with a suitable coat of inorganic substances such as manganese oxide (IV), alumina, silica gel, or graphene [34]. It can also be covered by organic compounds such as MIPs, surfactants, and divinylbenzene. Table 5 shows the main applications for MNPs.

In one example [31], magnetic solid-phase microextraction (MSPME) with CoFe_2_O_4_ NPs for the determination of cadmium in oyster and water samples using flame atomic absorption spectrometry (FAAS) was performed. The synthesis of cobalt nanoferrite was performed by the co-precipitation method with the addition of sucrose. Such a procedure allowed the formation of Co Fe_3_O_4_ with an average size of 5.0 nm and with magnetic properties. The optimized analytical methodology is simple and robust. It allows the determination of the Cd with a LOD of 0.24 mg L^−1^ The analysis of oyster samples was performed with satisfactory relative recoveries (93.6–102%) with RSD (%) less than 5.6.

**Figure 4:**
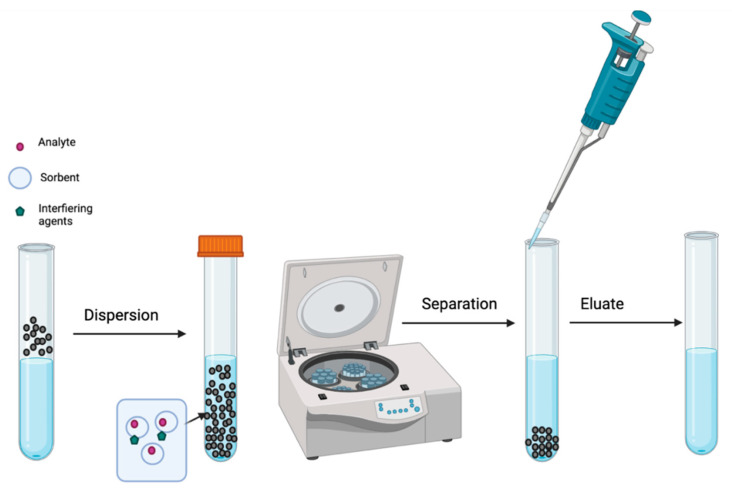
DSPE procedure is based on the dispersion of a solid adsorbent in a sample composed by a solvent and the target analytes. The sorbent can interact with the target analytes and could be easily separated using only centrifugation or filtration.

Another technique in this area is dispersive solid-phase extraction (microextraction) (DSPE/DSPME), which consists of the dispersion of a solid sorbent in a sample formed by a solvent and the target analytes that will be extracted. One of the advantages of this procedure is that the sorbent can interact directly with the target compounds and may be easily separated from the sample by centrifugation or filtration [42]. Moreover, DSPE is rapid and requires low solvent consumption [43]. The DSPE procedure is described in Figure 4, while Table 6 presents the main analytical methodologies, sorbents, and applications used in DSPE.

A very good example of such a type of extraction was presented by Tuzen et al [30]. In this research, the polystyrene-polyricinoleic acid copolymer containing silver NPs (AgPSrici) was created and applied in the separation of molybdenum ions from different aqueous and food samples during the DSPME approach. The high adsorption capacity of the AgPSrici adsorbent was found as 170 mg g^−1^ for Mo(VI) ions. An additional advantage is that the AgPSrici adsorbent can be used at least 60 times for the adsorption and desorption steps without any decrease in its sorption properties. In order to shorten the extraction time, the methodology was supported by the vortex-assisted extraction and centrifugation process. The present DSPME procedure was characterized by such benefits as fast, simple, low cost, eco-friendly, high enhancement factor, sensitive, and selective. Analytical data of the method were calculated and the LOD, LOQ, and RSD were 0.022 µg L^−1^, 0.066 µg L^−1^, and 2.9%, respectively.

A very good analytical performance was obtained by the application of magnetic NPs in DSPME. A very good example of such an application was presented by Mohammadi et al. [44], who used a magnetic dispersive solid-phase microextraction technique (MDSPME) based on the chitosan–iron oxide magnetic nanocomposite (CS@Fe_3_O_4_) as a sensitive and inexpensive technique for the preconcentration and simultaneous determination of selected pesticides in fruit and vegetable samples before determining with GC–FID. The limit of detection for the determination of selected analytes was in the range of 0.3–1.0 ng mL^−1^. The proposed methodology presented a high recovery percentage in the range of 95.8–106.0 and low RSDs (≤5.0) for the determination of selected pesticides in the cucumber and orange samples.

**Table 6:** Information on the DSPE procedures used for analytical applications.

DSPE Technique	Brief Description	Sorbents Used	Applications
Quick, Easy, Cheap, Effective, Rugged, and Safe (QuEChERS) method.	QuEChERS is based on the dispersion of salts to isolate a wide variety of analytes.	Primary Secondary Amine (PSA) Octadecyl Silica (C_18_) Graphitized Carbon Black (GCB).	Environmental analysis (Isolation of OCs, Ops, and carbamates pesticides from seeds and soils) [45] Pharmaceutical and clinical analysis (identification of xenobiotics in human blood and tissue) Isolation of food contaminants. Mycotoxins in breakfast cereals, fruits, and vegetables [46]
Dispersive micro-Solid Phase Extraction (D-μSPE)	The approach of conventional DSPE allows the possibility to reduce the amount of sorbent used for the extraction procedure (in the milligram range).	Micro materials such as PSA, C_18,_ Graphitized Carbon Black (GCB), MIPs. Nanostructured sorbents such as: Nanoparticles (NPs) such as Au and metallic oxides (SiO_2,_ Al_2_O_3,_ TiO_2,_ ZrO_2_) [47] Carbonaceous materials. Layered double hydroxides. Metal–organic Frameworks (MOFs) Hollow porous MIPs [48,49].	
Magnetic Solid-Phase Extraction (MSPE).	MSPE uses a magnetic adsorbent in a solution. Analytes can be absorbed on the surface to be isolated and eluted with appropriate solvents.	Magnetic particles combined with silica and carbonaceous materials. The most popular materials used for MSPE are Carbon Nanotubes (CNTs), Activated Carbon (AC), Graphene (GP), and Graphene Oxide (GO) [50].	

**Figure 5:**
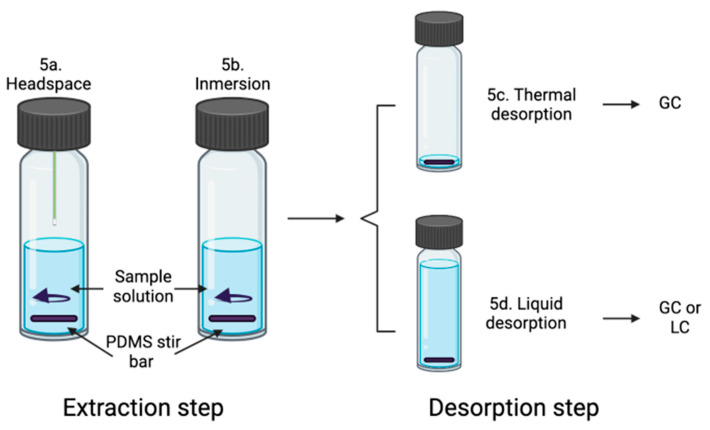
The main steps for the adsorption of the sample in SBSE can be performed in two different extraction modes: immersion (**5a**) or headspace (**5b**) modes. The extraction procedure is followed by thermal desorption if the separation is carried out by gas chromatography (**5c**) or liquid desorption if the sample is analyzed by liquid chromatography or GC (**5d**).

Another important extraction technique that applies nanosorbents as materials for extraction processes of environmental contaminants is stir bar sorptive extraction (SBSE), which is a novel technique that miniaturizes sample preparation and reduces the solvent amount used for this procedure. In addition, SBSE couples the extraction procedure with the analysis, providing higher sensitivity, better recovery of the analyte, and a reduced sample amount for the analysis [51]. The analytes can be extracted from the matrix into a nonmiscible liquid–liquid extraction [52]. In Figure 5, the SBSE procedure steps are shown. One of the main advantages of SBSE is that it has good applications in the recovery of trace levels of volatile organic compounds (VOCs) and semi-volatile compounds. The main applications of SBSE are described in Table 7. There is a wide variety of materials used for sorptive extraction; one of the most common sorptive extraction phases is polydimethylsiloxane (PDMS), among the two others commercially available, which are polyethylene glycol (PEG) and polyether sulfone (PES)-coated stir bars [53]. However, it is universally acknowledged that the development of new coatings for stir bars is desired, which affects the selectivity, dynamics, and recovery of the SBSE-based method. Here, many of the NPs are introduced into stir bar coatings to improve the extraction efficiency. In such "home-made" stir bars, the adsorption process is mainly involved; however, both absorption and adsorption processes sometimes exist simultaneously. The examples of NMs that are applied to create the new coatings or to modify other materials of stir bars are carbon materials (graphene [54], graphene oxide [55], carbon nanotubes [56], metal–organic frameworks (MOFs) [57,58], and molecular imprinted polymers [59]). Many times, different kinds of NMs are mixed together to enhance the extraction process. A good example of such a solution was presented by Fan et al. [60]. The authors applied MIPs in SBSE as a special coating to improve the selective extraction capability for propranolol (PRO) from urine samples. However, due to the incompatibility in aqueous media and low adsorption capacity of MIPs, which limit its application to stir bars in aqueous samples, a water-compatible graphene oxide (GO)/MIP composite-coated stir bar was prepared. The new water-compatible GO/MIP-coated stir bar was characterized by good mechanical strength and chemical stability. Moreover, due to the polymerization of MIP in the water environment, the recognition ability of prepared stir bars in aqueous samples was improved. In addition, the adsorption capacity for analytes of interest was also increased by the addition of GO in MIP pre-polymer solution. The prepared, new stir bars were applied for the sorptive extraction of PRO coupled to HPLC-UV. The LOD of the proposed procedure was about 0.37 gL^−1^, while the RSD was found to be 7.3%.

Another example of the mixing of two materials was presented by Wu et al. [61]. In this research, a vanillin-MIP-coated SBSE based on the magnetic field-induced self-assembly of multifunctional Fe_3_O_4_@polyaniline NPs for HPLC-UV analysis of the vanilla flavor enhancer in infant milk powders was presented. The complexes were adsorbed on the surface of a magnetic stir bar under magnetic induction, and the coating of vanillin-MIPs was generated by the one-step copolymerization based on the crosslinking of ethylene glycol dimethacrylate. The molecular imprinting stir bar showed superior selectivity and fast binding kinetics for vanillin, and was used for the enrichment of vanilla-flavor enhancers in infant milk powders. The results measured by HPLC-UV exhibited a good limit of detection of 2.5–10.0 ng mL^−1^, and the recoveries were 82.1–98.9% with RSD < 7.2%.

**Table 7:** Information on main applications of SBSE.

Type of Analysis	Matrix	Application/Compounds Measured
Water analysis	Sea water River water Lake water Wastewater	Phenolic, amine-based, acid, and apolar estrogens in wastewater. Pesticides (OCPs, carbamates, OPPs, and pyrethroids) in aqueous solutions. PBDEs and PCBs in river water [62].
Soil analysis	Living plants Fungi Soils	Flame retardants (PCBs and PBDEs) in soil. Pesticides in living plants and soils [63] VOCs (chemical signals) in plant material.
Food analysis	Wine Beer Fruits and vegetables Juices Baby food Herbal teas	Pesticides in fruits and vegetables [64] PAHs in beverages such as green tea, wine, and water. Monoterpenes in fruits and vegetables. Preservatives in beverages. VOCs in wine and fruit.
Biological samples	Biological tissue Blood plasma Whole blood Blood serum Urine	Isolation of methylmercury in human and fish tissues [65] Xenobiotics metabolites in human urine [62].

## 4. Nanomaterials as Sorbents for Extraction Used for Environmental Samples

What is well known is that the collection of the sample is the first stage in the analytical procedure for the identification of chemical compounds from real samples. One of the choices for this step can be the sorptive extraction method, and, here, nanomaterials are widely applied [4,66]. For most of the samples, a pre-treatment procedure is much often required such as centrifugation, Soxhlet, microwave, or ultrasonic extraction and liquid extraction to separate the analytes from the matrices. After that, analytes can be preconcentrated by the application of the sorptive extraction method. Such a solution permits the improvement of the selectivity and sensitivity of the eventual analytical methods for final determination and quantification. Nanomaterials are very often applied for the determination of pollutants such as volatile organic compounds (VOCs) and their by-products [67], pesticides, polychlorinated biphenyls (PCBs), polyaromatic hydrocarbons (PAHs), and endocrine-disrupting chemicals (EDCs) in different kinds of environmental samples [68]. The NMs used in the appropriate extraction technique are dependent on the type of sample. It can be found in the literature that MOFs are applied in the process of air sample preparation for the determination of formaldehyde [69], aromatic hydrocarbons, C2-C5 volatile fatty acids, phenols, indoles [70], toluene, *n*-hexane, butanone, methanol, dichloromethane, and *n*-butylamine [71]. Carbon nanotubes (CNTs) such as graphene-packed needle trap devices [72], single-walled carbon nanotubes-SiO_2_ (SWCNTs-SiO_2_) [73], and multi-walled carbon nanotubes-SiO_2_ (MWCNT-SiO_2_) [74] have been utilized for the determination of various VOCs in the air in the SPME/needle trap technique.

In the solid and sediment samples, such pollutants can be presented: PAHs, pesticides, PCBs, and EDCs. Due to the occurrence of these compounds at trace-level concentrations, isolation and pre-concentration with the application of a proper sorbent material are also required and can be performed by the use of NMs such as MWCNT-OOH [56,75], MOFs [76,77], and polyaniline-modified zeolite NaY [78].

Nanomaterials-based solid-phase extraction techniques have effectively replaced liquid–liquid extraction methods for the determination of organic pollutants in water samples. This is mainly due to the clean-up, low volume of solvents used, high throughput, broad selectivity, and simple automation. In the literature, such NMs as nanostructured polyaniline-ionic liquid composite [79], cetyltrimethylammonium bromide-modified zeolite NaY [80], polyaniline nanowires [81], and MWCNTs [82] have been examined for the extraction of the different kinds of pollutants that occurred in water samples. It needs to be noted that the sensitivity of the NM-based extraction techniques conjugate with analytical techniques are comparable or even superior to those of other extraction techniques in terms of many validation parameters, including the linear range, accuracy, precision, and LODs.

## 5. Conclusions

During recent decades, various NMs have been introduced and applied as promising media for the adsorption of many organic pollutants such as PCBs, PAHs, EDCs, and other VOCs from different types of environmental samples and others. The nanometer-sized particles are characterized by a large surface area that provides high selectivity, high adsorption capacity, enrichment capability, fast adsorption, and better mechanical and chemical stability as sorbents in extraction techniques such as SPE and SPME. All of the parameters of appropriate NPs used as a sorbent medium in extraction techniques strongly depend on the specific NMs used (Table 8). Not only were the adsorption efficiencies of such a sample treatment solution greatly improved in comparison to commercially available products, but the analytical parameters including sensitivity, selectivity, repeatability, and reproducibility were also improved. In addition, other benefits were achieved such as the reduction in time required for the overall procedure, as well as the reduction in the sample volume. Furthermore, the low cost, eco-friendly, easy modification, and easy separation approaches make NMs more valuable for the pretreatment and extraction of specific analytes from different matrices. However, it must be mentioned that much effort is still required to expand the practical application of NMs for the isolation and preconcentration of trace-level pollutants that occur in environmental samples. In addition, it would be great if universal procedures exist for the manufacturing of NMs characterized by the same morphology, size, and surface chemistry. Future efforts should also be made for the efficient coupling of NM-based sorptive extraction techniques with other types of advanced methodologies as such developments could impact on the wider utility of these procedures in the area of environmental sample analysis. It is also expected that other novel sorbents such as MOFs and functionalized graphite can be used as robust, high-capacity, and fast-functioning sorbents for some of the extraction techniques and can be commercially available. Depending on the nature of NPs, they can be applied to extend the applicability to more polar solutes; and if the manufacturing of NPs can be performed cost-effectively, it would result in the procedure of choice for many extractions, even making single-use devices such as disposable stir bars possible.

## Figures and Tables

**Table 1:** Information on NMs classified in different classes.

Type of NMs	Examples	Notes	Ref.
Carbonaceous nanomaterials	Graphene quantum dots	The nanostructured carbonaceous materials have shown exceptional behavior in extracting and preconcentrating trace-level organic contaminants prior to analysis.	[7]
	Carbon nanotubes (CNTs)	Provide high chemical stability, more surface area, small pore size, hollow structure, and easy modification compared to conventional adsorbent materials. The adsorption efficiency of CNTs also depends on their purity, surface area, functional groups present on the surface, adsorption sites, and experimental parameters.	[7]
	Graphene nanoribbons	Display a finite bandgap when their width is less than 10 nm, and their electronic behavior changes from semiconductors to semimetals as their width increases.	[8]
	Graphene	High surface area, low cost, delocalized pi-electrons, easy modification. More effective adsorbent than CNTs and fullerenes.	[9]
	Graphene oxide	The active sites of GO make easier the synthesis of composite material.	
Magnetic nanoparticles (MNPs)	Core–shell Fe_3_O_4_ polydopamine NPs	Nanosized particles having super magnetic properties, high surface reactivity, large surface area, high adsorption ability, and easily adjustable temperature.	[10]
	Iron oxide NPs	The adsorption capacity of MNPs can be enhanced through physical or chemical modification with complexing agents/organic compounds.	
Ion-imprinted polymer nanoparticles (NIPs)	Fe_3_O_4_@SiO_2_@IIP NPs; Ni–Fe_3_O_4_@IIP; Pb-IIP	Highly selective adsorbents for the preconcentration and extraction of template ions in a complex matrix. The selectivity of NIPs as adsorbents is based on the ligand specificity toward the metal ion, coordination geometry, coordination number of the ions, charge, and size.	[11,12]
Silica nanoparticles (SiNPs)	Hybrid amine-functionalized	Have a high surface area, and different diameter and size of particles, and is easily modified due to the presence of silanes.	[13]
	titania/silica nanoparticles	The limitation of silica nanoparticles includes narrow pH band (pH 2–8), and chemical and thermal instability.	
NPs based on metal–organic frameworks (MOFs)	TMU-8; TMU-9; MOF-199 consist of iron oxide nanoparticles-immobilized 4-(thiazolylazo) resorcinol (Fe_3_O_4_@TAR)	The pore shape and size make the MOFs highly selective and ideal adsorbents. Are easily dispersed and extracted from a sample mixture with the use of a magnet.	[14]

**Table 2:** Information on the SPE sorbents.

Group		Examples	Description/Uses
Inorganic oxides	Silica-bonded phases	Octyl-bonded silica Butyl-dimethyl-bonded silica Graphene oxide (GO) Amino-based silica	Have adsorbent properties with a high number of contact surfaces areas [18].
	Alumina-based packing	Alumina-A Alumina-B Alumina-C	The most common applications of inorganic oxides are the isolation of polar pesticides from fats and oils [15,17,18].
	Synthetic magnesium silicate (Florisil^®^)	LC-Florisil Envi-Florisil	
Low-specificity sorbents	Silica-bonded Sorbents	Siloxane-bonded sorbents 3-cyanopropyl, 3-aminopropyl	Commonly used for isolation of pollutants from an aqueous solution.
Low-specificity sorbents	Porous polymer sorbents	Copolymers of styrene and divinylbenzene.	
	Graphitized carbon blacks and porous graphitic carbon		
Compound-specific and class-specific sorbents	Immunosorbents [19]		Based on molecular recognition by antibodies [2].
	Molecularly imprinted polymers (MIPs)		Used as synthetic analogs of immunosorbents.

**Table 3:** Main modes in which SPME can be performed.

SPME Mode	Description	Types of Compounds that Can Be Analyzed
Direct immersion (DI-SPME)	Fiber coated with a sorbent directly exposed to the matrix. Analytes must go directly from the matrix to the sorbent.	Isolation of volatile compounds from biological matrixes.
		Complex biological matrixes: blood, urine, hair.
Head Space SPME (HS-SPME)	Fused silica-fiber coated with adsorbent exposed directly in headspace above sample.	Preferred for semi-volatile compounds.
		Soil, food, and biological samples.
Protective membrane SPME	DI-SPME used together with a protective membrane, which is used to prevent the diffusion of high-weight molecules in the extraction phase.	

**Table 5:** Main applications of MNPs.

	Application	Examples
Biological samples	Isolation of ribonucleic acids (RNA) and desoxyribonucleic acids (DNA) from biological fluids, viruses, and bacteria. Protein purification Isolation of organic and inorganic compounds from complex biological fluids such as blood plasma, blood serum, urine saliva.	Estrogens in plasma samples from pregnant women [35]. Steroid hormones in human urine samples [36]. Fenitrothion in human plasma and urine samples [37].
Food samples	The binding of biomolecules such as antibodies or aptamers on the surface of MNPs causes the isolation process of different contaminants in food.	Acetanilide herbicides in green tea samples [38]. Pyrethroids pesticides in rice, wheat, and corn samples. Hg (II) in fish samples [39].
Environmental samples	Isolation of ions and heavy metals in water, soil, and air samples Identification and quantification of persistent organic pollutants (POPs), such as organochlorine and organophosphate pesticides, PBDEs, PCBs, pharmaceutical products, PAHs, and phthalates.	AHs in seawater samples. Carbamate pesticides in river and rice field water samples. [40] Heavy metals in the water of river and lakes: Cu (II), Ni(II), Cd(II), Pb (II), Mn (II) [41].

**Table 8:** Comparison and characteristics of specific NPs used as a sorbent in extraction techniques.

Types of Nanosorbents	Advantages	Limitations	Stability	Selectivity	Reuse Time	Extraction Time (min)	Recovery [%]	Applications	Ref.
Graphene	Large specific surface area; provides more adsorption sites and loading capacity; low synthesis cost; the technique does not require the application of pressure during the extraction procedure (mainly in the MSPE case)	The use of a material with large surface area may create large backpressure problems (for SPE, microextraction by a packed sorbent (MEPS), and on-line methods)	Good chemical stability under strong-acid, strong-base, and high-salinity conditions; stable mechanical properties	Specific selectivity depending on the modifiers/components used.	50	50	80–113	Applied in SBSE: extraction of PAHs, organochlorine pesticides (OCPs), amino acids, and fluoroquinolones in environmental, food, and biological samples. Applied in MSPE: extraction of naphthols; Applied in SPE: Determination of tetracyclines in milk; determination of metals in environmental samples.	[54,83,84,85,86,87]
Graphene oxide/graphene oxide frameworks (GOFs)	Good adsorption capacity for organic compounds, especially medium and polar compounds; the presence of functional groups in GO can interact with metals and organic analytes by electrostatic and hydrogen bonding; GOFs have a high specific surface area, multiple electronic properties, and high porosity; GO has a good dispersibility in most solvents and is easily lost during the extraction process.	The extraction efficiency is mainly low, and the kinetics is also quite slow (50–90 min)	Good mechanical stability	Specific selectivity depending on the modifiers/components used.	50	50	75–115	Used in SPE: extraction of fatty acids in seeds, insecticides in flowers; Used in MSPE: extraction of OCPs in honey and fruit juice, Determination of PAHs in oil samples; determination of metals in aqueous samples; Used in SBSE for extraction of amphetamine and methamphetamine in biological samples	[88,89,90,91]
Carbon nanotubes (CNTs)	CNTs have a tubular structure, high specific surface area, and hydrophobic surface, which are suitable for adsorbing nonpolar target analytes.	The specific surface area will vary according to the number of layers. It is characterized by insoluble properties in aqueous solutions and organic solvents. The extraction kinetics is usually slow.	High mechanical stability.	Through surface modification, e.g., amino, carboxyl and PEG, their surface-active groups can be increased to promote the EE of polar target analytes.	30	30–180	70–120	CNTs have been used as SBSE coatings to separate and enrich organic pollutants from different matrices (such as food, biological, and environmental samples) through π–π interaction, van der Waals forces, and hydrophobic interaction. Used in SBSE for the extraction of OCPs, herbicides, and PAHs in water and environmental samples, extraction of naproxen in biological samples	[56,92,93,94]
MOFs	MOFs have available crystal properties, adjustable ultra-high porosity, large BET surface area (2000–7000 m^2^/g) and pore volume, uniform porous structure, abundant functional groups, and excellent photoelectric properties. Different metal centers and ligands are applied to produce MOFs as molecular building blocks, which results in a suitable flexibility for modifying physical and chemical features. Exhibit superior tunability of pore size and functionality	MOFs are sensitive to moisture, the structure is damaged owing to the occupation of water molecules.	High thermal, chemical, and mechanical stability	MOFs can be modified with chemical groups that uniquely affect the overall selectivity and sensitivity of the extraction process.	10–140	30–90	85–110	Hydrophobic interaction and π–π interaction enable MOFs to adsorb aromatic organic pollutants well. Applied in SBSE for benzylpenicillin in milk and biological samples; in HF-SBSE for phthalates in baby food; in SPE for the extraction of hormones from serum samples; in SPME for extraction of drugs in biological fluids	[56,57,95,96,97]
MIPs	High adsorption efficiency can be observed through various mechanisms, including π–π interactions between the delocalized π–electron system of the target analytes and the aromatic rings, the sorbents, and hydrophobic interactions. A further increase in the affinity toward the target analytes can be achieved through functionalization of the MOF sorbent.	Low adsorption capabilities that may arise in aqueous media.	Stability can be modified by application of additives (e.g., graphite oxide and nanoparticles, etc.) and/or the deposition of the MOF on substrates.	It has specific recognition and selective adsorption capabilities for specific target molecules (template molecules) and their analogs. Exhibit strong affinity toward small organic compounds.	50–120	10–180	80–110	Applied in SBSE for extraction of estrogens and glucocorticoids from water, milk, and urine samples, for diclofenac extraction in seawater and commercial tablet samples; applied in vortex-assisted d-SPE of parabens from environmental waters, cosmetic creams, and human urine samples. Applied in MSPE for extraction of OPCs in urine, phthalate esters from plasma samples; applied in SPME for extraction of naproxen and its metabolites in biological samples.	[49,98,99,100,101,102]

## Data Availability

Not applicable.
